# Introducing double fortified salt in social safety net programmes in Madhya Pradesh and Gujarat in India: Success factors, challenges and lessons learned

**DOI:** 10.1111/mcn.13646

**Published:** 2024-06-05

**Authors:** Becky L. Tsang, Shilpa Deshpande, Mini Varghese, Sakshi Jain, Daniel Lopez de Romana, Manpreet Chadha

**Affiliations:** ^1^ Food Fortification Initiative Atlanta Georgia USA; ^2^ Independent Consultant Hyderabad Telangana India; ^3^ Nutrition International New Delhi India; ^4^ Nutrition International Ontario Ottawa Canada

**Keywords:** anaemia, double fortified salt, food fortification, implementation, India, iron deficiency, micronutrients

## Abstract

Double fortified salt (DFS; with iron and iodine) was introduced in social safety net programmes (SSNPs) in Madhya Pradesh (MP) and Gujarat states in 2018. Nutrition International (NI) provided critical support for the intervention. An impact evaluation in MP found high DFS uptake, exceeding 90%. Conduct a process evaluation of the DFS programmes in MP and Gujarat states to identify success factors, challenges, and recommend considerations for scale‐up. Twenty‐eight qualitative interviews were conducted with NI staff, national and state level government officials, and DFS producers in 2022. Enabling environmental factors included national‐level support for food fortification, consensus that anaemia was essential to address, and institutional trust in NI for technical assistance. In programme implementation, the primary challenges were reports of black specks in DFS and the darkening of food cooked with DFS. NI supported the government in improving handling practices, ensuring a regular and stable supply, introducing quality monitoring efforts and launching targeted behaviour change communication (BCC) campaigns regarding the value of DFS. Long‐term implementation of the programmes is a weak point, as DFS production is more expensive than iodised salt, there is no existing market outside of institutional demand, and BCC must be long‐term, high‐quality, and requires resourcing for continued high uptake among SSNP beneficiaries. Strong government buy‐in and technical support along the supply chain to address quality issues and beneficiary acceptance were key factors for the successful introduction of DFS. Comparative studies of DFS programmes should be conducted to improve confidence in the success factors that lead to high DFS uptake.

## Key messages

- Double fortified salt (DFS) in Madya Pradesh demonstrated the potential to address iron deficiency if there is high acceptance and use by consumers.
- Overcoming sensory issues with DFS will require ongoing resources in behaviour change communication and consumer subsidies to make DFS more attractive and price‐competitive with other products available in the marketplace.
- Strong government buy‐in is necessary for funding and readily accessible technical support along the supply chain to address quality issues and beneficiary acceptance.
- Comparative studies of DFS programmes should be conducted to improve confidence in the success factors that lead to high DFS uptake.

## 1 INTRODUCTION

While the addition of iron through fortification has been practiced globally since the 1940s (Global Fortification Data Exchange, 2022a), the inclusion of iron to foods has been limited to cereal grains (wheat and maize flour, more recently rice) (Global Fortification Data Exchange, 2022b), with some success also in powdered milk (Hurrell, 2021). Given the various constraints in India to fortify cereal grains (Bhatnagar & Kanoria, 2020) and milk (G & Gupta, 2014), salt appears poised as a more advantageous food vehicle to fortify with iron to address high anaemia prevalence in India (57%) (International Institute for Population Sciences & ICF, 2022). Recent evidence indicates that iron deficiency was the strongest predictor of anaemia in an analysis of the Comprehensive National Nutrition Survey 2016–18 (Scott et al., 2022).

However, fortifying salt with iron faces distinct challenges (Diosady et al., 2002, 2019; Drewnowski et al., 2021). To address these needs, Nutrition International (NI; then Micronutrient Initiative) partnered with the University of Toronto's (U of T) Department of Chemical Engineering and Applied Chemistry to develop a new form of DFS that utilised an encapsulated and masked form of iron compound, encapsulated ferrous fumarate (EFF).

India's experience with DFS, in DFS production and past pilot projects and programmes over the last decade, is well‐documented (Moorthy & Rowe, 2021; Shields & Ansari, 2021). More recently, from 2018 to 2022, NI provided technical assistance (TA) to the state governments and industry partners in two states, Madhya Pradesh (MP) and Gujarat, to introduce DFS in two social safety net programmes (SSNPs) – under the Public Distribution System (PDS) in MP and Integrated Child Development Services (ICDS) in Gujarat. An overview of both programmes is in Supporting Information: Text. State representative estimates of iron deficiency and iron‐deficiency anaemia are not available, but the anaemia prevalence among women of reproductive in both MP (53%) and Gujarat (65%) (International Institute for Population Sciences & ICF, 2022) exceeds the World Health Organization's (WHO) cut‐off for a ‘severe public health problem’ (World Health Organization, 2011). While the MP DFS programme has been quantitatively evaluated for health impact before and after the introduction of the intervention, there has been no qualitative evaluation of the programmatic implementation of these two programmes.

The objective of this evaluation was to build evidence for effective and sustained DFS programmes by exploring the actions and processes involved in introducing DFS to the state SSNPs in MP and Gujarat and describing the successes, challenges and lessons learned from stakeholders in India.

## 2 METHODS

### 2.1 Data collection

The process evaluation utilised a semistructured interview guide, adapted for each type of key stakeholders involved in the DFS programme decision‐making and implementation process (NI technical support staff, national and state government officials, and DFS producers) (Supporting Information: Interview guides). Key actors identified by NI included 9 NI staff (current and former) involved in supporting national and state‐level DFS efforts, 23 national and state government officials, and 2 DFS producers. The in‐depth interview guide focused on two areas of activities: (1) how an enabling environment that set the stage for the introduction of DFS into SSNPs was established and (2) the programme‐level activities to be implemented by the state governments to distribute DFS to beneficiaries and supported by NI through TA.

Two co‐investigators (B. L. T. and S. D.) who led the development of the interview guides also held the interviews. The interviews were divided among the co‐investigators to prioritise local language needs and in‐person interviews where possible. S. D. interviewed government officials and DFS producers in Hindi or English (depending on the interviewee's preference) at their place of work unless they requested a virtual interview, and B. L. T. interviewed NI staff in English virtually on Zoom. While NI contracted S. D. and B. L. T. to conduct the process evaluation, neither S. D. nor B. L. T. are, or have been in the past, NI staff or involved in any DFS programme implementation. Interviewees provided verbal consent. The in‐person interviews were conducted in Bhopal and Gandhinagar, and interviews were conducted in July–August 2022. Interviews were recorded with an audio device or using Zoom's ‘record’ function, transcribed in the original language, and identifying information was removed. Interviews held in Hindi were not translated. Only anonymized, transcribed interviews were shared with NI co‐authors.

### 2.2 Data analysis

Interview transcripts were analysed by the author who had conducted them. NI co‐authors did not participate in data analysis to avoid potential conflict of interest. Coding was deductive and structural. Interviews were first coded based on interview guide topics and questions. Subsequently, the coded data was further analysed to identify programme activities, steps, successes, enabling elements and challenges or lessons learned as per the Programme Impact Pathway (PIP) framework proposed by Larson et al. (2021).

The evaluation was approved by [IRB approval # removed for anonymization]. Quotes referenced here have been modified only minimally to improve readability.

## 3 RESULTS

Eight potential government respondents refused an interview. Of these, two were national, and six were state government representatives. Their reasons included discomfort with an in‐depth interview format, instead delegating a subordinate to participate in the interview, and/or unavailability due to retirement and other personal reasons. Of the 17 who agreed to the interview, 4 were national technical experts, 11 were state government representatives and 2 were salt producers. With these respondents, 11 in‐person and 15 virtual interviews (including NI staff) were done. The in‐person interviews were conducted in Bhopal and Gandhinagar, and most interviews were completed in July 2022.

### 3.1 Establishing an enabling environment

**Figure 1:**
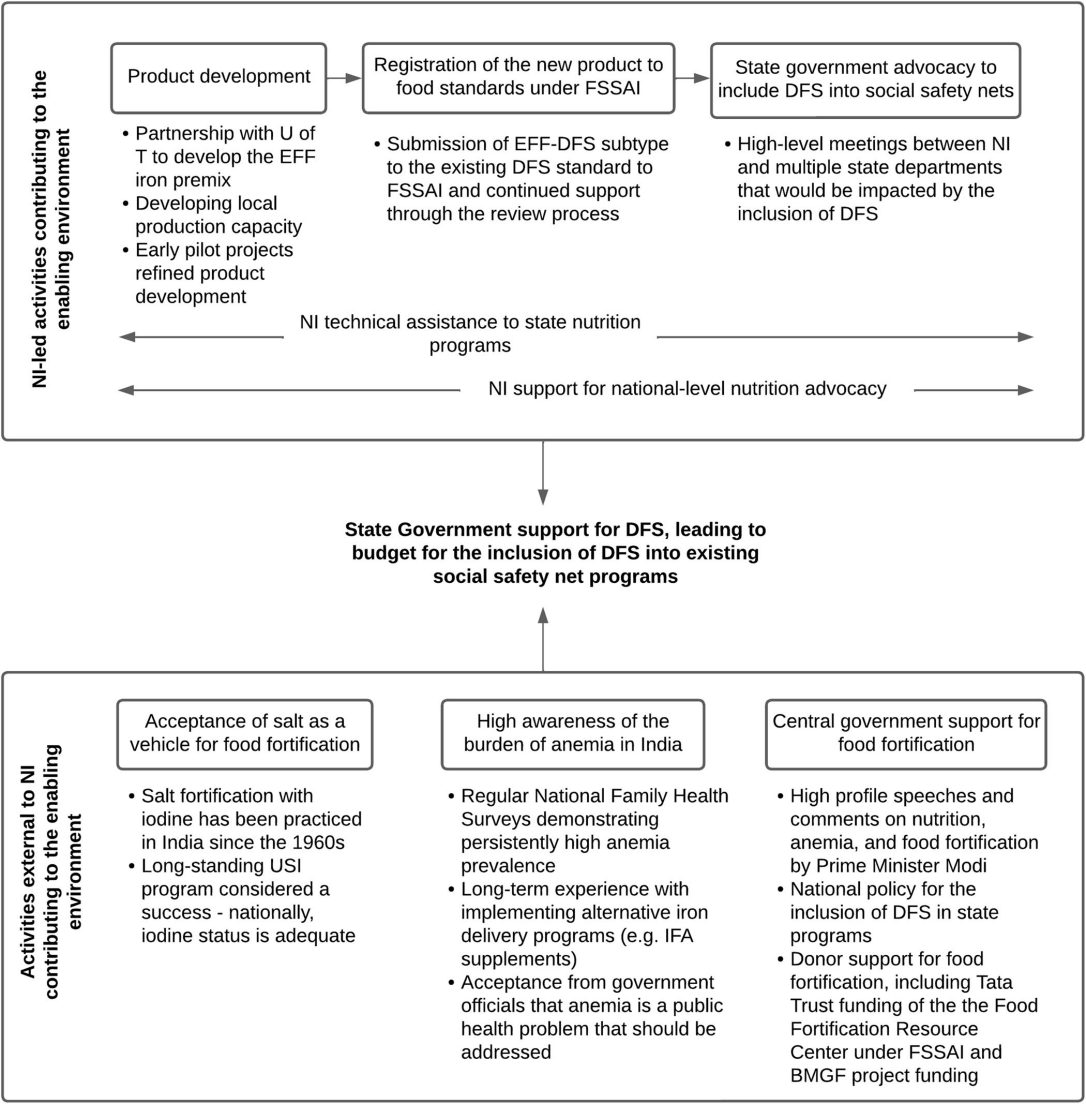
Elements attributed by interviewees as contributing to the enabling environment for DFS in social safety net programmes in Madhya Pradesh and Gujarat. DFS, double fortified salt; EFF, encapsulated ferrous fumarate; FSSAI, Food Safety and Standards Authority of India; HQ, headquarters; IFA, iron and folic acid; NI, Nutrition International; U of T, University of Toronto; USI, Universal Salt Iodisation.

An overview of the elements identified through interviews considered to contribute to a successful enabling environment for the inclusion of DFS into SSNPs is in Figure 1. Elements could be classified as NI‐led (either activities directly related to DFS or general nutrition TA) and elements outside of NI's influence. When added together, they appeared to be influential factors in garnering the financial support of MP and Gujarat states to implement DFS in their SSNPs. Challenges and lessons learned within each activity and an overview of DFS in the MP and Gujarat state programmes are provided in Supporting Information: Text.

### 3.2 Analysis: Implementation of DFS against a PIP framework

**Figure 2:**
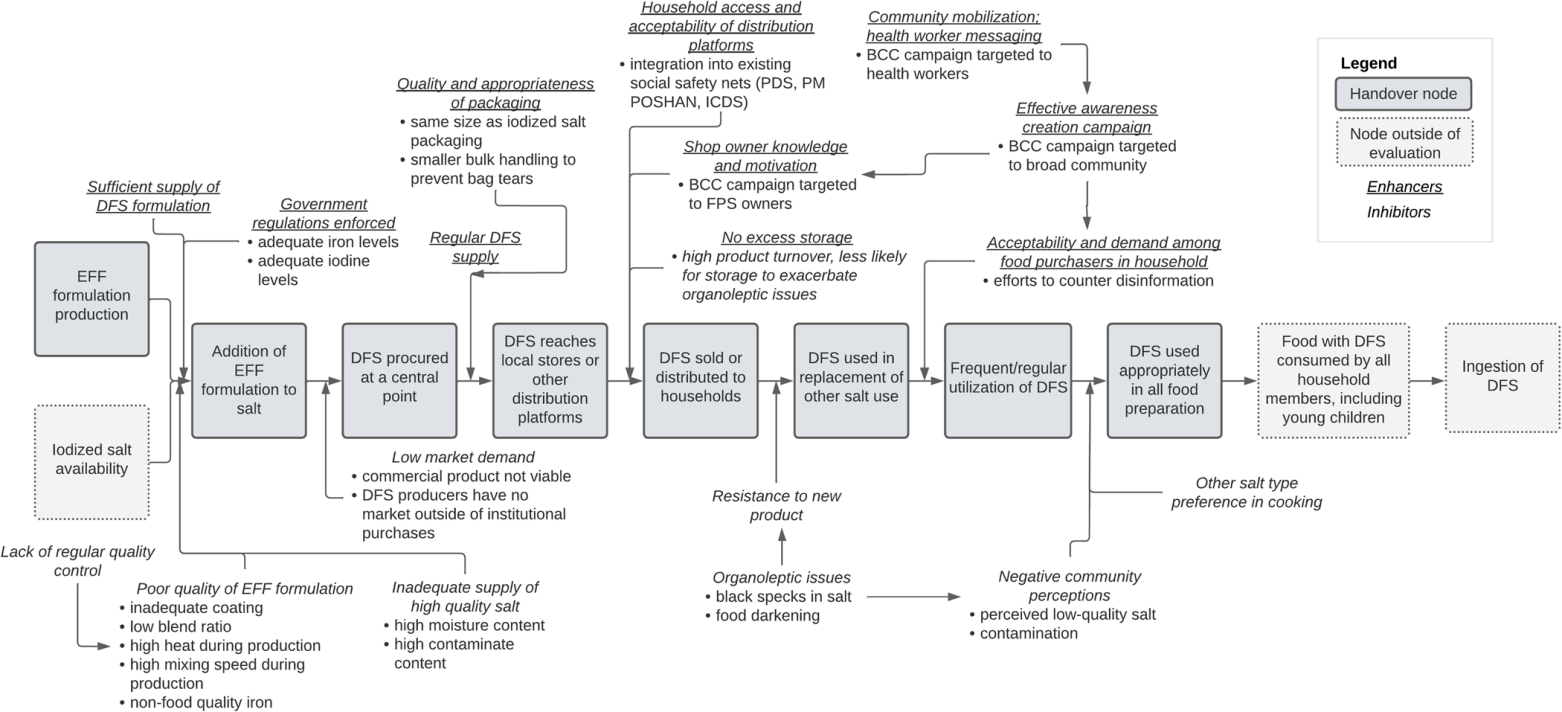
Modified Programme Impact Pathway for elements identified in the MP and Gujarat DFS programmes. Modified from Larson et al. (2021). BCC, behaviour change communication; DFS, double fortified salt; EFF, encapsulated ferrous fumarate; FPS, fair price shop; ICDS, Integrated Child Development Services; PDS, Public Distribution System; PM POSHAN, Pradhan Mantri Poshan Shakti Nirman (school meals programme).

Programme activities, successes, challenges and lessons learned referenced in interviews are categorised in ‘handover nodes’ along the PIP. See Figure 2 for PIP modified from Larson et al., referencing the handover nodes and enhancing and inhibiting elements discussed in the MP and Gujarat DFS programmes. The adapted PIP is not state‐specific because many elements are applied to both MP and Gujarat. Where state programmes differed in implementation (e.g., different targeted audiences for behaviour change communication [BCC]), they are described below.

#### 3.2.1 Iron premix production

##### Inhibitor – Lack of regular quality control, leading to poor quality iron premix (at EFF production level)

Producers of EFF must be registered with the Food Safety and Standards Authority of India (FSSAI), but it is not clear what requirements or processes they must follow. Interviewees repeatedly raised that the lack of a standard for the EFF was a quality gap that affected the entire DFS programme downstream. The FSSAI guidebook on Food Fortification for Food Safety Officers only references quality control measures to measure the presence of iron and iodine in the salt (Food Fortification Resource Center, 2022). Beyond meeting basic eligibility criteria to respond to a tender, winning bidders were primarily selected based on the lowest price (referred to as L1 bidders).

With an emphasis on low cost, extremely low installed capacity utilisation by EFF producers, and no product standards to adhere to, there was very little room for EFF producers to market their products based on quality parameters. This resulted in cost‐cutting measures that led to the production of low‐quality EFF, such as increasing the concentration of iron within an EFF granule (e.g., from 15% to 18%), which allowed for a lower blending ratio of EFF to iodised salt (e.g., 5% instead of 7%).

However, because an EFF granule must still maintain a certain density and volume to homogeneously blend with iodised salt granules, using a higher iron concentration required reducing the use of other ingredients. Where less encapsulation was used, it increased the risk of iron interacting with iodine, resulting in iodine losses. Where less colour masking (titanium dioxide) was used, the EFF granules may have been visibly distinguishable within the blended DFS. Producers also used nonfood grade ferrous fumarate in EFF, a consumer safety concern, to cut costs; an interviewee felt that this practice was indicative of the likelihood of employing other cost‐cutting measures that would impact sensory characteristics. The quality‐related shortcomings of DFS have also been described elsewhere (Jadhav & Mannar, 2021; Shields & Ansari, 2021). Interviewees noted that under a controlled laboratory environment by the University of Toronto, there are no sensory issues with EFF. But when translated to the market dynamics of remaining competitive for government DFS tenders, compromising production practices became common practice.

Interviewees also stated that the black specks in EFF‐DFS were of initial concern, as beneficiaries and even a Minister raised complaints of ‘sand’ in the salt, adulteration, or poor quality salt being distributed. Despite these sensory issues, ultimately there was >90% DFS uptake at MP's end line survey (Nutrition International & AMS, 2021), suggesting that the black specks from EFF‐DFS could be overcome through BCC.

Poor salt quality was briefly raised in interviews as a constraint to the supply of DFS. Because the DFS standard requires a higher quality salt than specified for iodised salt (Shields & Ansari, 2021), the limited amount of salt produced in India meeting DFS standard requirements could be a future barrier to scale‐up. To address this, one interviewee suggested that more resources should be devoted to determining whether DFS truly required higher quality salt or if the addition of DFS could be done with the same quality salt used for iodisation.

#### 3.2.2 Addition of iron premix to salt

##### Enhancer – Government regulations enforced

Although poor quality EFF was an issue in both states, efforts to monitor DFS for other parameters such as iron/iodine content, moisture and NaCl levels were considered successful. NI conducted these lab tests every month; in MP, the Civil Supplies Corporation Limited (CSCL) also took random samples from every order for the state food lab to test iron and iodine levels. In Gujarat, the CSCL set up a Food Research Laboratory to sample each DFS batch for iron and iodine content. If samples failed the tests, penalties included held payments, returned batches, fines, or supplier blocklisted. In Gujarat, in particular, long testing turnaround (17–20 days) combined with frequent failing samples that required suppliers to re‐submit new batches for testing affected the regularity of the DFS supply. Throughout the programme in Gujarat, the quality monitoring process did become more streamlined, with assay turnaround improving to 24–48 h.

[Laboratory tests] really helped the government to give instant feedback to the vendor on each of the batch, take remedial action. There were many times when the payment of the vendor was stopped, that, ‘Unless you give us the good quality salt, we're not going to do the payment’.

However, enforcing quality parameters was not considered a success by all interviewees. Another interviewee's perspective was that despite efforts, the quality of the DFS did not change over the life of the programme, stating that the only thing that has changed is that ‘we have become a bit wiser, [now we are able to say] that the problem is at the production point or problem is at the EFF production’. Although quality monitoring was considered an essential activity, it appears not to have been entirely successful for all parameters – being more challenging to scale up and maintain throughout the project.

##### Inhibitor – poor quality of iron premix (at the salt processor)

After EFF has been produced and purchased by a salt processor, the salt processor blends EFF with iodised salt. Ribbon blenders, a standard piece of blending equipment used for the addition of iodine to salt, are also used to add EFF to the iodised salt. However, the interviews described flaws when processes and equipment used for iodised salt were applied to DFS, which has also been described elsewhere (Shields & Ansari, 2021). NI training to reduce damage at this point of production included educating processors to modify practices to reduce blending speed (and thereby heat) – but the damage could not be eliminated entirely. The automated packaging process used by many salt processors also generated more abrasive contact than ideal for EFF, and the only potential solution for this was incorporating manual packaging for DFS. These adaptations to improve existing salt processor practices to incorporate DFS with reduced damage ultimately added time and reduced efficiency to produce DFS.

##### Inhibitor – High price leading to low market demand (for a commercial product)

Although both MP and Gujarat's programmes distributed DFS under brand names linked to the SSNPs (*Vanya+* in MP, *Sattva and Kalpataru* in Gujarat), interviewees indicated that the lack of expansion from SSNPs to commercial DFS products prevented improvements to DFS quality because producers saw no incentive to improve the product outside of institutional demand. Notably, ‘all [salt processors] have tried [to develop their own branded DFS product]’, but none have been successful in launching DFS commercially.

Although the DFS was marketed with a mascot and brand name, none of these brand names were linked to the original salt processor (and processors would vary depending on changes in the winning bidder). As a result, any consumer perceptions of lower quality were associated with the state SSNP rather than the salt processor.

There is no risk, no benefit – nothing that the salt processor would gain for [producing higher quality DFS]…. today, this quarter, they are supplying the DFS to say, MP; next quarter it would be somebody else. There's no incentive for that salt processor to set up a … dedicated production facility or [to] invest [in] quality control/quality assurance to ensure that their final product is really good.

Although NI trained salt processors to improve the quality of DFS by modifying existing equipment, salt processors were highly unlikely to pursue improvements that required capital investments.

Without giving salt processors [the incentive] to set up [optimal production] facilities, or a vision that tomorrow, the market might improve and then they will have demand from the open market, they're not going to invest. The supply for the PDS and other social safety net programs are not helping that cause because of the L1.

Furthermore, at a fixed margin, DFS was being supplied to PDS for individuals below the poverty line; salt processors did not feel that a high‐quality product was needed for distribution in a SSNP.

The thing that is there in their mind is [that] this [product] goes for social safety net programs where it's poor people who [receive] this double fortified salt … if a product is available at, say, one rupee, then not much value is added in terms of how the appeal should be or the crystal size or the free‐flow‐ness of the salt. It's a cheap product that is available.

##### Inhibitor – insufficient supply of high‐quality salt for blending with EFF premix

Poor salt quality was briefly raised in interviews as a constraint to the supply of DFS. Because the DFS standard requires a higher quality salt than specified for iodised salt, the limited amount of salt produced in India meeting DFS standard requirements could be a future barrier to scale‐up. To address this, one interviewee suggested that more resources should be devoted to determining whether DFS truly required higher quality salt or if the addition of production of DFS could be done with the same quality salt used for iodisation.

#### 3.2.3 DFS procured at a central point

##### Enhancer – Quality and appropriateness of packaging (during handling)

Another point of damage occurred when the state FCS godown/warehouse workers handled bulk DFS packages. Individually packaged 1 kg DFS bags were combined for transportation and handling into one bulk bag containing 50 retail bags. These bulk bags were handled with large metal hooks to transport them as needed. When these hooks punctured the 1 kg bags, the exposure to moisture (combined with the pre‐existing issues of poor EFF production and damage during blending) caused ‘entire bags of DFS’ to turn black.

NI provided technical support to the government to modify its handling practices (reducing the bulk bag size to 25 kg to allow manual handling instead of hooks) and training *godown/warehouse* workers on these modified handling practices. Although they were relatively simple changes that required minimal communication time, the messages needed to be constantly and continually communicated. The effort was described as a ‘huge amount of training’, which required ‘continuous hand‐holding and capacity‐building support with warehouse staff’.

##### Enhancer – Regular DFS supply/no excess quantities stored

NI supported states to calculate the required DFS volumes to ensure an uninterrupted supply. Although interviewees described some supply delays in Gujarat due to failed sample tests, on the whole, there was no significant gap in DFS delivery to the SSNPs. This was important because if rations are not received in a given month, beneficiaries are entitled to a double ration the next month. As there is no secondary market for an oversupply of DFS (Cyriac et al., 2022), any extended storage of DFS in the household can lead to greater sensory issues or use of DFS for nonconsumption purposes, such as feed for farm animals. In fact, the MP end line survey suggested that the PDS salt ration was not enough to fulfil household cooking needs – at end line, only 80% of households indicated that the ration was sufficient for their needs (Nutrition International & AMS, 2021).

#### 3.2.4 DFS reaches local stores or other distribution platforms

##### Enhancer – Household access and acceptability of distribution platforms

The significance that DFS was included within heavily subsidised/free SSNPs cannot be underestimated. Interviewees attributed the high uptake of DFS through PDS in MP specifically to the steep financial incentive of PDS‐provided DFS at 1 INR/kg compared to iodised salt. Considering access was integrated into the supply chain of an existing delivery platform (FPS), beneficiaries did not need to change their behaviour to find DFS. Even compared to crude salt, DFS from the PDS was more affordable. At least in MP, because making changes to the PDS is such a massive endeavour, proposing a phased approach by starting with a subset of districts in a particularly vulnerable population (tribal areas) was a more manageable initial commitment by the government. Not only was the programme state‐led through the provision of budget and human resources necessary to implement, but an interviewee cited how important it was that the state also incorporated DFS into existing data systems, such as the state's management information system and biometrics system for PDS uptake and offload.

As NI resources were limited to TA, all implementation expenses came from state government budgets, including the actual procurement of DFS and dissemination of BCC materials. With the cost of DFS at 2–2.5 INR/kg higher than iodised salt, introducing DFS into the PDS of 20 districts in MP meant allocating an additional 686 lakh INR (822,816 USD) annually for the additional cost of DFS alone – excluding the time and resources required for modifications in the PDS supply chain to incorporate DFS, print or disseminate BCC materials, and train BCC intermediaries such as FPS owners, and healthcare workers (Accredited Social Health Activists [ASHAs], and *Anganwadi* workers).

Readily available, close‐contact technical support by NI at all administrative levels (district, state and central government) was considered essential to ensure that the state government received the support needed to implement DFS in SSNPs.

One interviewee stated ‘the success of any scheme is possible if we work in all these fronts and closely with the government…’ and described fielding frequent requests from government officials to respond to questions or assist with developing necessary materials.

Since not many state governments were procuring DFS, NI's support in developing technical quality norms for the tender was particularly helpful in Gujarat. As a government official stated:

The good thing about [NI] is that we get feedback whether something is working well or not working well, whether the supply is reaching or not, we get to know everything frankly.

However, state and NI interviewees repeatedly mentioned one policy gap as inhibiting broader household access to DFS throughout the country: the lack of a directive from the central government that DFS must be used in SSNPs. Although this directive exists for PM POSHAN (D.O. No.5‐5/2011‐MDM‐1‐1 EE.5, 2011) and ICDS (No. 5‐4/2011 ND/Tech, Mandatory Use of Double Fortified Salt [DFS] in National Programmes—ICDS, 2011), it is not clear how well this is enforced, and no mandate exists for PDS.

#### 3.2.5 DFS sold or distributed to households

##### Enhancer – Community mobilisation; health worker messaging/shop owner knowledge and motivation

Both the MP and Gujarat programmes included BCC targeted to beneficiary intermediaries: FPS owners and ASHAs in MP and ASHAs and *Anganwadi* workers in Gujarat. In MP, FPS owners were educated about awareness of iron and iodine deficiencies and trained to disseminate information about DFS. However, not all FPS owners participated in these training – of those interviewed for the end line survey, 60% had participated in NI‐led training. Of those trained, the local Food and Civil Supply Officers felt that the training ‘improved the flow of information from FPS owners to the beneficiaries’ (Nutrition International & AMS, 2021).

In Gujarat, DFS began in a targeted demographic – women and children within the ICDS programme – and distributed as a take‐home ration for women and within a cooked meal for children aged 3–6 years. As such, BCC targeted *Anganwadi* workers through satellite communication sessions organised by the state government. Consumer‐targeted awareness was still important since there was a take‐home ration, but the beneficiaries could be reached through *Anganwadi* workers at the centres at the time of distribution. After DFS was scaled to include PM POSHAN and ICDS programmes in MP, it became essential that BCC efforts also extend to these targeted users.

#### 3.2.6 DFS used in replacement of other salt use

The end line survey in MP indicated uptake (reported purchase and use of DFS through DFS for 6 months) above 90% (Nutrition International & AMS, 2021). Although a similar baseline and end line evaluation was not conducted in Gujarat by NI due to the supply of DFS being complicated by the COVID‐19 pandemic, interviews with state officials reported that the Indian Institute of Public Health in Gandhinagar conducted its monitoring efforts of the ICDS programme and 85%–90% of women and adolescent women receiving take‐home rations reported using DFS.

#### 3.2.7 Frequent/regular utilisation of DFS

##### Enhancer – Effective awareness creation campaign, leading to acceptability and demand among food purchasers in households

NI developed a BCC campaign using formative research to understand how to reach beneficiaries, their intermediaries, and the correct messaging and language (Nutrition International, 2017; Public Health Foundation of India, 2017). In MP, because DFS was incorporated into a programme (PDS) where DFS was used in the home, it was also necessary to develop mass consumer BCC materials that were broad in reach, and several different modalities were utilised, including radio, print media and videos. Consideration had to be made for reaching consumers who were illiterate or had limited education. Marketing techniques were employed, such as having a ‘mascot’ for DFS (Purna in Gujarat, Lali in MP).

A significant amount of effort was also required for responsive or defensive communications materials to explain what the black specks were and to address ‘fake news’ about DFS – such as videos to contradict claims that DFS will not dissolve in water. More than one interview stated that BCC campaigns and training on DFS in Gujarat should have occurred before the product's launch, which could have prevented misconceptions about DFS and improved acceptance.

#### 3.2.8 DFS used appropriately in all food preparation

##### Enhancer – [prevention of] Negative community perceptions

DFS also required different storage and cooking practices to reduce potential sensory changes compared to crude or iodised salt. NI created BCC targeted at beneficiary households instructing consumers to store the 1 kg bag of DFS in a separate container to protect it from moisture and, for a lower likelihood of food discoloration, add it at the end of cooking rather than during. It is not clear how successful this messaging was at changing consumer behaviour. Still, interviewees felt that any discoloration due to poor storage was thought to be low, given the high salt turnover at the home.

##### Inhibitor – Organoleptic issues leading to negative community perceptions

Despite the product quality improvement efforts and BCC efforts previously described, sensory issues persisted, affecting the initial acceptance of DFS, and limited uptake in a small proportion of the population at the end line. These issues were limited to the visual appearance of the DFS and in some foods (such as yellow lentils, potato curry, etc.) cooked with DFS – ‘black specks and food turning black’. There were no reported changes in the smell or taste of the salt or foods cooked with DFS.

## 4 DISCUSSION

Introducing a new food or intervention into established, high‐coverage SSNPs like PDS, PM Poshan, and ICDS is a long‐term, complicated endeavour requiring the buy‐in and efforts of multi‐sector stakeholders. There is no accepted approach to achieving significant systemic changes within these programmes, and there are no known previous assessments of what has or has not worked to introduce new foods or interventions.

However, understanding the factors that contribute to such an enabling environment would open up significant opportunities in a country like India, where SSNPs are expected to reach not just half of the population but also the most nutritionally vulnerable and often the hardest to reach through large‐scale food fortification.

However, once a new food is introduced, there needs to be more programmatic understanding of how to integrate operations into the existing SSNP. Although several states have already integrated iodised salt into their PDS supply chain, introducing DFS required additional adaptations or sensitivities. Outside of this report, there has only been one other assessment of the implementation of a DFS programme – a fidelity of implementation (FOI) analysis conducted on the first large‐scale DFS distribution in PDS project in Uttar Pradesh (UP) to understand better how well the programme implementation operated as intended and provide further context to the end line impact evaluation (Cyriac et al., 2022). Other analyses of DFS implementation have also focused on the UP project (Jadhav & Mannar, 2021). Below, we discuss the successes and weaknesses of launching DFS, both from the enabling environment and implementation.

### 4.1 Enabling environmental successes

An essential element of the enabling environment was the government's (national and state level) support for food fortification, DFS specifically. Lack of political will due to perceived conflicts with other health agendas has prevented DFS projects in other countries from even launching (Moorthy & Rowe, 2021). Although political will and the source of such support can be amorphous to pin down, interviews suggested that at least two key factors played a role: a popular prime minister with previous experience and support for food fortification leading the central government and ownership of both programmes at the executive level of MP and Gujarat's state governments – crucial given that both states needed to fund DFS in its entirety with state budgets.

An influential fortification champion to continue pushing the food fortification agenda forward is often cited as a success factor for programmes (Martorell & de Romaña, 2017). Although the stakeholder interviews conducted for this work could not provide insight into how or why Prime Minister Modi supports food fortification, it is possible that his earlier experiences with wheat flour fortification as the Chief Minister in Gujarat made a long‐term impact.

Martorell and de Romaña (2017) also point out that domestic institutional research capacity was a feature of successful fortification programmes in Latin America. In India, interviews also highlighted the importance of having long‐term, nutrition and health data generated by an accepted government source to show that high anaemia prevalence was a stagnant health concern despite existing nutritional efforts and socioeconomic gains across the country. The National Institute of Nutrition played a supportive role in Gujarat during expert consultations. However, domestic research institutions can also have a significant negative influence. Questions over the degree to which iron deficiency contributes to anaemia have been raised by prominent Indian scientists regarding the current or proposed use of multiple concurrent iron interventions (Kurpad et al., 2021; Kurpad & Sachdev, 2022).

### 4.2 Programme implementation successes

As described in the UP FOI (Cyriac et al., 2022) and case studies (Jadhav et al., 2019), there are several differences between the programmes in UP and those in MP and Gujarat. The key difference between UP and MP is uptake. In MP, 90% of beneficiaries purchased DFS for the last 6 months, and of those, 99% reported daily use (Nutrition International & AMS, 2021). The UP programme found high coverage (74% purchased at least once) but with low use (35% reported partially using DFS in cooking) (Cyriac et al., 2020). Although this work is not intended to be a comprehensive comparison between UP, MP and Gujarat and why the uptake proportions were so different across programmes, it is worth pointing out barriers identified in the FOI that differed from details described in interviews and could have played contributing factors.

#### 4.2.1 Differences in BCC strategies that may have led to higher community mobilisation and final beneficiary acceptance

Sensory issues with DFS were experienced in all three states, but the beneficiary response to these sensory issues differed significantly. The FOI found that training for FPS owners and ASHAs had limited effectiveness (Cyriac et al., 2022) and FPS owners reported low motivation to sell DFS because of these issues. BCC to beneficiaries also did not seem to penetrate, as individuals were still unaware of the presence of iron in DFS or had negative perceptions (Cyriac et al., 2022). On the other hand, this work found that interviews consistently attributed high DFS uptake in MP and Gujarat to BCC efforts. Without a thorough analysis of the BCC strategies and differing other factors between UP, MP, and Gujarat, it is impossible to attribute the differences in uptake to BCC alone. Nevertheless, UP and MP had vastly different outcomes in beneficiary perception. In MP, 85% of respondents preferred DFS over other types of salt (Nutrition International & AMS, 2021) (not measured in Gujarat).

#### 4.2.2 Lack of bundling salt with other commodities

PDS in UP ‘bundled’ or combined the purchase of DFS with other higher‐demand commodities, such as grains (Cyriac et al., 2022). Although this successfully led to high coverage (proportion of households purchasing DFS), counterintuitively, it did not lead to high utilisation of DFS for cooking. FPS owners said that this led to excess storage at the household, and beneficiaries repeated that they were ‘forced’ to take the salt, possibly leading to or exacerbating the perception of DFS as a poor‐quality commodity (Cyriac et al., 2022). Conversely, interviews in this process evaluation reported consistent DFS supply to households as one of the major successes. Lack of excess storage at the household, leading to increased salt turnover and prevention of further sensory issues related to extended storage, could have been another factor in high uptake by beneficiaries in MP and Gujarat.

### 4.3 Programme implementation weaknesses

Interviews identified two main weaknesses – (1) the continued sensory issues with DFS and (2) the need for high‐quality, maintained BCC across the product supply chain to counter negative perceptions from the sensory issues. Sensory issues were the primary barrier to uptake in UP (Cyriac et al., 2022; Jadhav et al., 2019) and MP and Gujarat and continue to drive research into alternate formulations of DFS.

One interviewee estimated that BCC efforts needed to continue for at least a couple of additional years in Gujarat, considering the reach required to communicate to the new PM POSHAN and PDS programmes. Scaling up DFS from 20 districts in MP (and BCC was only done in 5 districts, to begin with) to all 34 districts in MP will mean that BCC will need to reach another 35 million beneficiaries – which does not include efforts to educate intermediaries such as FPS owners or ASHAs. Scaling from SSNPs that provide DFS in cooked meals to home rations (e.g., from PM POSHAN to PDS) are particularly in need of BCC because although DFS will come at a steep discount, beneficiaries still have the choice of whether to purchase. As an interviewee put it, ‘[In PDS in Gujarat], definitely a [BCC] strategy will have to be strong and comprehensive’. The MP evaluation suggests that if other state governments can replicate such BCC strategies, high uptake by beneficiaries is possible, and most importantly, lower anaemia and iron deficiency prevalence.

However, the continued need for ‘intensive’ BCC implemented by state governments is connected to an overall enabling environment weakness. In the case of DFS in SSNPs, however, its sustainability relies solely on state governments continually prioritising funding DFS procurement, distribution, BCC and quality monitoring. The annual cost of subsidising DFS in MP alone (without the additional costs of increased training, BCC, and quality monitoring that are unique to DFS compared to iodised salt) was estimated to be 4102 lakh INR (5 million USD) in the 20 districts (Moorthy & Rowe, 2021).

As an interviewee stated, ‘it's not sustainable because right now [DFS] is only for the social safety net programs. Once [the government] or political party [change], the policy might change. And since it's not taken up by the open market, it's very difficult to continue…. The entire burden is basically on the state to finance [salt] procurement [in PDS], it's not getting funds from the national government’.

### 4.4 Application towards scaling up DFS across other programmes and states in India

Unless the central government announces nationwide mandatory use of DFS in SSNPs, scaling up DFS in India will require a state‐by‐state approach to introduce DFS. While the experiences in MP and Gujarat could provide a template to engage with state officials on the effectiveness or logistics of DFS implementation, state governments still need to choose to re‐allocate budgets to procure DFS. In MP, the additional cost to replace iodised salt in PDS with DFS represents 2.9% of the Food and Civil Supplies Department budget.

National DFS scale‐up may face similar challenges currently posed by the national introduction of rice fortification in PDS, particularly as iron is the specific nutrient where toxicity concerns have been raised (Kurpad et al., 2021). It is also possible that rice fortification itself could be a conflict for the scale‐up of DFS. Designing appropriate standards for the co‐fortification of multiple foods with the same nutrients will be essential to avoid over‐intake of nutrients such as iron.

Lessons learned from MP and Gujarat that would benefit other SSNPs planning to introduce DFS include (1) early, high‐quality BCC, developed to target multiple stakeholders – including FPS, ASHAs and the final beneficiaries; (2) quick adoption of newer, improved iron premix, when made available; (3) integration of DFS into existing SSNPs, systems and purchasing practices; (4) introduction of quality monitoring and reporting practices (and their maintenance and scale‐up) and (5) ready access to TA to support policy justifications, develop tenders and identify modifications or adaptations to the supply chain to maintain quality (but without adversely disrupting existing systems).

### 4.5 Study limitations

As this process evaluation was limited to implementation staff at the national and state levels and policy stakeholders, all programme perceptions described here may differ from the view of individuals directly interacting with end‐users or end‐users themselves. This report is intended to complement the findings of an end line evaluation in MP, which did assess end‐user feedback (Nutrition International & AMS, 2021). In UP, the commitment or perception of DFS in frontline individuals (FPS and ASHAs) and end‐users did differ from district level staff (Cyriac et al., 2022).

### 4.6 Further research needs

UP is the only other state that has introduced DFS in one of its SSNPs at a similar scale of distribution as MP and Gujarat but with a much lower DFS uptake. It would be of value to conduct a comprehensive comparative study of the three programmes to clarify further what worked, did not work, what may have been specific to the populations targeted in each programme, and what may be generalisable to the broader population.

## 5 CONCLUSION

Successful implementation of DFS in MP generated India‐based evidence that DFS can improve anaemia and iron indicators when distributed through a publicly run SSNP. High uptake in both MP and Gujarat states, despite continued reports of black specks in salt, is counter to past experiences in other Indian states or countries, where sensory changes to salt and foods cooked with DFS led to low utilisation or scrapped projects entirely. Immediate plans by both state governments to expand DFS within and across SSNPs will provide an opportunity to evaluate how well the ‘enhancers’ identified can be scaled up and sustained by the government.

However, for MP and Gujarat to serve as models for other states or global examples for DFS implementation, significant resources are required in areas not traditionally considered priorities within food fortification programmes – BCC and public subsidies. If sensory changes with DFS cannot be eliminated, then DFS programmes will require long‐term, comprehensive BCC campaigns that can combat negative messaging that the DFS is of low quality, contaminated, or causes adverse outcomes. Where alternate food vehicles can be carriers for iron and feasibly fortified, partners and governments should consider an analysis of the comparative value of each vehicle given their relative reach and cost–benefit ratio.

## AUTHOR CONTRIBUTIONS

Sakshi Jain, Mini Varghese, Daniel Lopez de Romana and Manpreet Chadha conceptualised the project in consultation with Becky L. Tsang. Becky L. Tsang and Shilpa Deshpande designed the research study and interview tools, with reviews from Sakshi Jain, Mini Varghese, Daniel Lopez de Romana and Manpreet Chadha. Becky L. Tsang and Shilpa Deshpande interviewed stakeholders and analysed data. All authors contributed to writing the paper.

## CONFLICTS OF INTEREST STATEMENT

Sakshi Jain, Mini Varghese, Daniel Lopez de Romana and Manpreet Chadha are employees of Nutrition International (NI). NI provides technical support to the central Indian, Madhya Pradesh and Gujarat state governments to implement the distribution of double fortified salt with iron and iodine (DFS) in social safety net programmes (SSNPs). Sakshi Jain, Mini Varghese, Daniel Lopez de Romana and Manpreet Chadha did not participate in interviews, data analysis, and NI authors only had access to anonymized transcribed interviews. Mini Varghese was a key stakeholder for the introduction and scale up of the intervention in India and therefore was included as a key informant in this process evaluation. Mini Varghese's perceptions and opinions of the project were not reflected in the development of this manuscript. The remaining authors declare no conflict of interest.

## Supporting information

Supporting information.

Supporting information.

## Data Availability

The data that support the findings of this study are available on request from the corresponding author. The data are not publicly available due to privacy or ethical restrictions.
