# Supplementary material for: Introducing double fortified salt in social safety net programmes in Madhya Pradesh and Gujarat in India: Success factors, challenges and lessons learned
Source: Matern Child Nutr. 2024 Jun 5;22(1):e13646. doi: 10.1111/mcn.13646 (PMC12647980; doi:10.1111/mcn.13646)
Supplement: Supplementary file 1 — Supporting information. [file MCN-22-e13646-s001.docx]

Introducing Double Fortified Salt in Social Safety Net Programs in Madhya Pradesh and Gujarat States in India: Success Factors, Challenges, and Lessons Learned

**Supplemental text**

Activities led by Nutrition International

**Product development**

Although fortifying salt with the ferrous sulfate form of DFS had been proposed by the National Institute of Nutrition (NIN) in India as early as 1975 (Rao & Vijayasarathy, 1975), there were concerns of sublimation, leaving the iron in place but compromising the public health impact of decades of salt iodization (Diosady et al., 2019). The NI-U of T iron premix was made of a ferrous fumarate compound encapsulated with hydroxypropyl methylcellulose and titanium dioxide to cover the color of ferrous fumarate and prevent direct exposure to iodine (Diosady et al., 2019).

Since EFF-DFS was launched, U of T has continued to pursue improvements to the formulation to reduce the likelihood of adverse sensory outcomes, such as black specks and discoloration of food cooked with EFF-DFS. In 2019, the University of Toronto proposed an extrusion methodology as an alternative to the fluid bed agglomeration method to produce encapsulated ferrous fumarate (EFF), which would result in more uniformly sized EFF particles and reduce damage to the exterior of the EFF particle (Diosady et al., 2019). Where the fluid bed methodology produced an EFF that was “popcorn-shaped” with irregular edges, extrusion allowed for a smoother, more uniform particle, allowing for a more even coating of the encapsulation and masking additive (Diosady et al., 2019). However, as EFF (and more broadly, DFS) methodologies improve, challenges arise in translating those improvements to commercial production - particularly if improvements to EFF production require new equipment or new staff training. Use of extruded EFF in MP and Gujarat’s DFS programs began between 2019 and 2020, but it was not clear at any given time what proportion of the DFS supply in MP and Gujarat was produced using the fluid bed methodology vs. extrusion. Even as of 2022, an interviewee felt that it was likely that both methods were still in use.

To address iron premix quality, NI submitted for consideration to FSSAI an EFF standard and this is under consideration by the agency as of September 2022. An interviewee expressed concern that while the EFF standard was under the review process with FSSAI, in the interim period beneficiaries would continue to experience black specks and discoloration in their food, causing the “confidence of the people on DFS [to] go down.”

**Product registration**

NI contracted a law firm to assist NI with submitting an application to the FSSAI to include the EFF formulation of DFS into the existing DFS standard. The pre-existing DFS standard had been issued in 2012 by Bureau of Indian Standards (FSSAI’s predecessor as a standards-setting agency) and only recognized the ferrous sulphate formulation of DFS. In 2016, an updated standard recognizing the use of EFF in DFS was released by FSSAI (Food Safety and Standards (Fortification of Foods) Regulations, 2016, 2017).

Although social safety net programs are implemented at the state level in India, the state governments rely on national guidelines for operationalization. FSSAI is the regulatory body responsible for developing food safety and quality standards, including fortified foods. FSSAI’s statutory powers include issuing guidelines for the accreditation of laboratories for food testing, providing scientific and technical support to the central government, and collecting/collating data on food consumption, contamination, and food safety risks (Food Safety and Standards Authority of India, 2022). Part of their role also includes the promotion of awareness about food safety and nutrition in India, which for fortified foods is done through the Food Fortification Resource Center (FFRC) under FSSAI. NI worked closely with FSSAI to develop the DFS standards that were released in 2016 (Food Safety and Standards (Fortification of Foods) Regulations, 2016, 2017), as well as technical documents on staple food fortification that were disseminated through FFRC. In 2020, NI also submitted to FSSAI a proposed specification for the EFF used in DFS, to provide guidance to states and producers to improve the quality of EFF-DFS and address continuing reports of adverse organoleptic effects of DFS.

Although the updated FSSAI standard was operationalized in 2016, interviews described the review process as “political”, and “unscientific” efforts to oppose EFF. In hindsight, one interviewee felt that NI’s status as a Canadian non-governmental organization added a negative lens of outsider influence; they suggested that partnering with an Indian academic institution for initial buy-in (and perhaps external validation) of EFF would have led to less national opposition and a faster submission process.

**Advocacy with state governments**

It is not clear exactly where initial support for DFS in MP began, although the importance of eventual ownership and adoption by the state’s executive branch (Chief Minister’s office) is clear. Implementation of DFS in MP was preceded by a series of advocacy meetings three years earlier by senior NI headquarters staff with multiple state level departments. However, state level interviews suggested that the initiative was proposed by a senior bureaucrat who at the time was directing the National Health Mission. After his transfer to the Department of Food and Civil Supplies, he continued championing the use of DFS to address high anemia prevalence in the state.

Although this bureaucrat may have taken a leadership role in pursuing DFS, interviews also pointed out the essential need for buy-in from multiple state departments to support DFS: as the state’s agency responsible for nutrition and health, the Department of Public Health and Family Welfare would need to agree that DFS was a suitable strategy to address issues of anemia and micronutrient deficiency, but several other government agencies also played essential roles, including: allocation of funds by the state’s Tribal Welfare Department; procurement and transportation by the state’s Civil Supplies Corporation Limited (CSCL); oversight of Fair Price Shops (FPS) by the Food Directorate, and for ICDS and PM POSHAN, implementation by the Department of Women and Child Development and Department of Elementary Education (in MP)/Department of Education (in Gujarat), respectively. Advocating with all affected departments was essential because much of the labor to implement these programs would be executed by - and hopefully adopted long-term - by these departments.

Another interviewee pointed out that the health departments played a role as initial heroes or champions of DFS, setting the tone for other departments’ perceptions and reactions to DFS, and ensured that DFS remained on the agenda and progressed towards the identified goals. Spending the time to properly identify and cultivate DFS champions in particular was continually pointed to in interviews:

“There is a need to identify champions. We often miss them. There are certain people who are part of the system, like [the] government system, and who [are] champions [for] DFS or fortified rice. We need to identify them, work with them and speak with others, their peers.

In Gujarat, interviewees more broadly credited state level support to advocacy and expert consultations from multiple actors. Specifically, the Department of Women and Child Development consulted with experts from the NIN, UNICEF, as well as NI, prior to the adoption of DFS within their program.

**NI-government collaboration outside of food fortification**

NI provides technical support to the Indian government on multiple levels – national, state, and district, across multiple health interventions. NI is not solely involved in fortification and also includes health systems strengthening, adolescent nutrition and vitamin A supplementation program. A factor in the state government’s reception of NI’s advocacy for DFS could have been facilitated by its longstanding collaboration with NI in not just fortification but other health initiatives as well.

NI has also participated in national level promotional and awareness activities for food fortification, which included DFS (under umbrella nutrition campaigns, such as *Swasth Bharat Yatra*, “A journey for healthy India”). These activities were also done in concert with FSSAI. Here again, NI’s prior interactions with Indian agencies and officials outside of DFS could have influenced government acceptance of DFS.

Enabling elements or activities external to Nutrition International

**Acceptance of salt as a vehicle for food fortification**

The familiarity and recognition of salt as a successful fortification vehicle was noted by one interviewee as a potential success factor in the acceptance of DFS as an intervention by states. Although there remain subpopulations with poor iodine status (Sareen et al., 2016), and needed improvements to the implementation of the national Universal Salt Iodization program (Krishna et al., 2022; Rah et al., 2015), salt iodization is credited with nationally adequate median concentrations across residence (urban/rural, region), wealth quintiles, and zones (Nutrition International et al., 2019), which mirror drops in iodine deficiency disorders, such as goiter prevalence (Ministry of Health and Family Welfare, 2022). As of the last NFHS-5 in 2019-21, iodized salt (iodized to any level) was available in 94% of households nationally (International Institute for Population Sciences & ICF, 2022).

**Central government support for food fortification**

However, regardless of state level support achieved through direct advocacy, interviewees recognized the role of very strong public support for food fortification in general by Prime Minister Narendra Modi’s central government. Mr. Modi, who became the Prime Minister of India in 2014, previously championed wheat flour fortification in 2010 as the Chief Minister of Gujarat. After Mr. Modi became the Prime Minister, he championed nutrition-focused initiatives such as *POSHAN Maah*, and in 2021 made the announcement that all social safety net programs must distribute fortified rice on a national basis by 2024. Interviewees agreed that without precedent from the central government on food fortification, the state level departments may not have been so accepting of introducing a new fortified food into their programs.

One interviewee hypothesized that Mr. Modi’s support for food fortification stems from national competition to have better nutritional indicators than neighboring South Asian nations such as Bangladesh and Sri Lanka. For comparison, the most recent statistics for anemia and iron deficiency anemia prevalence in Bangladesh are 26% (Institute of Public Health Nutrition et al., n.d.) and 4.8% (*World Health Data Platform: Prevalence of Anaemia in Pregnant Women (%)*, n.d.), respectively in non-pregnant, non-lactating women (NPNL); in Sri Lanka, 35.4% (*World Health Data Platform: Prevalence of Anaemia in Pregnant Women (%)*, n.d.) and 10.8% (Jayatissa et al., 2017), respectively in pregnant women - compared to 57% (national population) (International Institute for Population Sciences & ICF, 2022) and 21.5% (Scott et al., 2022) (adolescents aged 10-19 years) respectively.

**High awareness of the burden of anemia in India**

Interviewees pointed to the need to have government-accepted sources of data to demonstrate the public health need that DFS was intended to address. Fortunately, the NFHS captures state level anemia rates (among other health indicators) on a five-year cycle. Using these data, NI was able to demonstrate persistent anemia rates, despite existing efforts by the Ministry of Health and Family Welfare around dietary diversification and distribution and promotion of iron-folic acid supplements. Interviews with state level departments agreed that anemia was an important issue that should be addressed. Again, there have been high-profile statements made by the central government - in 2018, Prime Minister Mr. Modi called for an “anemia-free India” (Agencies, 2018).

However, government officials did express doubt whether DFS as an intervention could address anemia in India – much of the DFS experience being presented were from other countries. In response, NI built in a baseline and end line impact evaluation to the MP project to serve as the first effectiveness evidence of DFS in India. The end line evaluation found a 4 percentage point decrease in anemia prevalence and a 5 percentage point decrease in the prevalence of iron deficiency in intervention sites compared to control sites (Nutrition International & AMS, 2021). Although the overall prevalence of anemia remained high in the intervention sites (61%), the distribution of anemia severity post-intervention shifted from severe and moderate to mild anemia.

*Overview of DFS in state programs*

MP state program overview

Distribution of DFS in MP through the PDS program began in 2018, in 89 tribal blocks of 20 districts (out of 52 districts). NI’s technical support focused on five districts: Jhabua, Alirajpur, Badwani, Dhar, and Khargone. The learnings from those five districts were intended to trickle to the rest of the districts. The PDS allotment of DFS was 1 kg per beneficiary household every month (38), for which the beneficiaries paid INR 1 per kg of DFS (for reference, open-market price of iodized salt ranged from INR 10-50 per kg, depending on the brand (Nutrition International & AMS, n.d.)) and purchased the DFS at FPS, alongside other PDS commodities. The PDS in the remaining 32 districts distributed iodized salt. Unlike other states, MP had already begun distributing subsidized iodized salt in their PDS in 2012 - so in MP, the state only needed to fund the additional cost of DFS over iodized salt. The additional INR 2-3 per kg (total INR 825 lakh/per year for the 89 districts, or 1.003 million USD/yr.) was funded by the Tribal Welfare Department. No non-iodized/non-DFS salt was available at FPS, although raw/coarse salt was available in the open market for INR 5-10 per kg.

Based on the experiences of DFS in the PDS program, in 2022, the program was scaled-up by the Department of Health and Family Welfare to an additional 91 blocks of 17 high-priority districts (chosen based on high anemia and maternal mortality). After this expansion, nearly half of the state’s 353 blocks are expected to receive DFS.

In 2020, the use of DFS was expanded to other government social safety net programs in MP, where DFS would be used in cooked meals for beneficiaries: ICDS, PM POSHAN, District Hospitals, and Nutrition Rehabilitation Centers (in 51 of 52 districts across the state). From 2018-2022, 194,532 metric tons (MT) of DFS were distributed by the MP government to beneficiaries either through PDS or through one of the state-sponsored meal programs. Annually, an estimated 11 million individuals benefited from DFS through PDS; by the end of the DFS expansion to the other social safety net programs, annually 11.8 million individuals are estimated to benefit from consuming DFS in state-sponsored meals.

Gujarat State program overview

In Gujarat, DFS began with state-wide implementation but in a much smaller program than PDS, electing instead to introduce DFS in all 33 districts through ICDS. ICDS is delivered through 52,649 *Anganwadi* centers (AWC) (Chaturvedi et al., 2018) covering 1.9 million women of reproductive age (pregnant women, lactating women, and adolescent girls) and 1.45 million children aged 3-6 years. The DFS was used in meals served to children aged 3-6 years and whe women and adolescent girls were provided a monthly 1 kg take-home ration of DFS. In total, 80,000-88,000 MT of DFS were distributed from 2018-2022 and intended to reach 3.6 million registered beneficiaries (NITI Aayog, Government of India, n.d.). In total, the cost of purchasing both components of the ICDS program was INR 2,500-3,000 lakh per year (2.4-3 million USD).

Later on, with NI’s advocacy, the state government of Gujarat chose to expand the introduction of DFS to other social safety net programs, namely PDS in 33 districts; DFS has been available through the PDS in these districts since July 2022. Correspondingly, take-home rations in the ICDS will be scaled back, as DFS distributed through PDS is expected to cover the same beneficiaries. Eventually, DFS is expected to scale-up to the whole PDS beneficiary population of 7 million households, or 35 million individuals.

**References**

Agencies. (2018, September 11). PM calls for anemia-free India. *Millennium Post*. https://www.millenniumpost.in/nation/pm-calls-for-anemia-free-india-318183

Chaturvedi, A., Nakkeeran, N., Doshi, M., Patel, R., & Bhagwat, S. (2018). Determinants of micronutrient fortified blended food (Balbhog) consumption among children 6–35 months of age provided through the integrated child development services program in Gujarat, India. *Indian Journal of Community Medicine*, *43*, 97–101. https://doi.org/10.4103/ijcm.IJCM_103_17

Department of Food Civil Supplies and Consumer Protection. (n.d.). *Targeted Public Distribution System*. https://food.mp.gov.in/en/targetted-public-distribution-system

Diosady, L. L., Mannar, M. G. V., & Krishnaswamy, K. (2019). Improving the lives of millions through new double fortification of salt technology. *Maternal & Child Nutrition*, *15*(S3). https://doi.org/10.1111/mcn.12773

Food Safety and Standards Authority of India. (2022, December 27). *About FSSAI*. https://fssai.gov.in/cms/about-fssai.php

Institute of Public Health Nutrition, United Nation Children’s Fund (UNICEF), icddr,b, & Global Alliance for Improved Nutrition. (n.d.). *National Micronutrient Survey 2011-12, Final Report*. Ministry of Health and Family Welfare. Retrieved December 13, 2022, from https://www.unicef.org/bangladesh/media/4631/file/National

International Institute for Population Sciences, & ICF. (2022). *National Family Health Survey (NFHS-5), 2019-2021. India: Volume 1. Mumbai: IIPS.* International Institute for Population Sciences. https://dhsprogram.com/pubs/pdf/FR375/FR375.pdf

Jayatissa, R., Fernando, D. N., & De Silva, H. (2017). *National Nutrition and Micronutrient Survey of Pregnant Women in Sri Lanka*. Medical Research Institute in collaboration with UNICEF and World Food Programm.

Krishna, E., Pal, A., Khobragade, A., & Panigrahi, S. (2022). Vast gap in iodization from production to plate—Hurdles in achieving Universal Salt iodization in India. *Journal of Family Medicine and Primary Care*, *11*(3), 847. https://doi.org/10.4103/jfmpc.jfmpc_999_21

Food Safety and Standards (Fortification of Foods) Regulations, 2016, 11/03/Reg/Fortification/2014 (2017). https://ffrc.fssai.gov.in/standards

Ministry of Health and Family Welfare. (2022, February 8). *Status of Goitre or Thyroid Disorders in India*. https://pib.gov.in/PressReleasePage.aspx?PRID=1796440

NITI Aayog, Government of India. (n.d.). *Health and Nutrition: Practice Insights, Volume 2*. NITI Aayog. Retrieved December 30, 2022, from https://www.niti.gov.in/sites/default/files/2020-08/Practice_Insight_Vol_II.pdf

Nutrition International, & AMS. (n.d.). *Report on baseline survey for encapsulated ferrous fumarate (EFF) double fortified salt (DFS) project in Madhya Pradesh*.

Nutrition International, & AMS. (2021). *Program evaluation of demonstration project on reducing iron deficiency anaemia through provisioning encapsulated ferrous fumarate (EFF) double fortified salt (DFS) in selected districts of Madhya Pradesh: Findings from endline survey*.

Nutrition International, ICCIDD, & Kantar. (2019). *2019. India Iodine Survey 2018-19 National Report*. Nutrition International. https://www.nutritionintl.org/learning-resource/india-iodine-survey-2018-19/#:~:text=The%20survey%20revealed%20that%2076.3,the%20World%20Health%20Organization%20guidelines.

Rah, J. H., Anas, A. M., Chakrabarty, A., Sankar, R., Pandav, C. S., & Aguayo, V. M. (2015). Towards universal salt iodisation in India: Achievements, challenges and future actions: Universal salt iodisation in India. *Maternal & Child Nutrition*, *11*(4), 483–496. https://doi.org/10.1111/mcn.12044

Rao, B. S., & Vijayasarathy, C. (1975). Fortification of common salt with iron: Effect of chemical additives on stability and bioavailability. *The American Journal of Clinical Nutrition*, *28*(12), 1395–1401. https://doi.org/10.1093/ajcn/28.12.1395

Sareen, N., Kapil, U., Nambiar, V., Pandey, R., & Khenduja, P. (2016). Iodine nutritional status in Uttarakhand State, India. *Indian Journal of Endocrinology and Metabolism*, *20*(2), 171. https://doi.org/10.4103/2230-8210.176363

Scott, S., Lahiri, A., Sethi, V., de Wagt, A., Menon, P., Yadav, K., Varghese, M., Joe, W., Vir, S. C., & Nguyen, P. H. (2022). Anaemia in Indians aged 10–19 years: Prevalence, burden and associated factors at national and regional levels. *Maternal & Child Nutrition*, *18*(4). https://doi.org/10.1111/mcn.13391

*World Health Data Platform: Prevalence of Anaemia in Pregnant Women (%)*. (n.d.). Retrieved December 13, 2022, from https://www.who.int/data/gho/data/indicators/indicator-details/GHO/prevalence-of-ana emia-in-pregnant-women-(-)
