# Supplementary material for: Introducing double fortified salt in social safety net programmes in Madhya Pradesh and Gujarat in India: Success factors, challenges and lessons learned
Source: Matern Child Nutr. 2024 Jun 5;22(1):e13646. doi: 10.1111/mcn.13646 (PMC12647980; doi:10.1111/mcn.13646)
Supplement: Supplementary file 2 — Supporting information. [file MCN-22-e13646-s002.docx]

**Process evaluation of the introduction of double fortified salt in pilot districts in Madhya Pradesh and Gujarat, India**

**In-depth Interview (IDI) with Government Officials**

**Date:** [date]

**Prompts**

- At start:
  - Read consent
  - Ask respondent if the interview can be recorded
  - Ask if there is an obligation at the top of the hour
- At end:
  - Is there anyone else that they think I should speak with?
  - Thank them for their time.

**Section – I: Background Information**

1. Government Official Name ______________________________________________

Designation _______________________________________________

Department _______________________________________________

1. Role within project ______________________________________________
2. Time frame involved ______________________________________________

**Section – II: Topic Guide for IDI**

1. **Background**
2. Can you please tell me about your Department’s role within the DFS implementation project?

(Has this role changed since the project began? If yes, why?)

1. What is your role/ responsibility in the DFS implementation project?

(Has this role changed since the project began? If yes, why?)

1. Who else did you work closely with on this project? (Individuals/Designation/Department)

(What were their responsibilities?)

1. Can you please describe a timeline of how the project in [state] rolled out?
2. **Activities/Tasks (Note – questions for specific sections only asked of individuals who had roles/activities related to those sections. E.g. a staff member who did not interface or work on advocacy with policymakers would not be asked questions under that section)**

Design of DFS introduction and implementation

1. What were the reasons for choosing DFS for implementation through the Public Distribution System/ICDS in Madhya Pradesh/Gujarat?
2. How were the intervention areas selected? (criteria) How was DFS introduction rolled out? (timelines, areas)
3. What kind of approvals were required to introduce DFS in the PDS/ICDS? How long did it take to receive the approvals?
4. What difficulties, if any, did you have in obtaining approvals for DFS introduction? How were these resolved?
5. What is the financial allocation for DFS? Is it likely to continue/sustain in future?

DFS procurement

1. What is the procurement process for DFS?
   1. Is it different from the procurement process for iodised salt? If yes, how is it different and why were these changes made?
   2. How was the tendering process put in place for DFS? What kind of support was required?
   3. What parameters were identified for selecting salt manufacturers - manufacturing process, capacity, price, frequency of supply, storage of stocks and how were these parameters identified
   4. How did the difference in price of iodised salt and DFS impact decision making and the procurement process?
   5. What was the response to the tendering process? Were any revisions required?
2. What are the quality criteria considered for DFS and its packaging? Were these criteria easily met? Did they have to be changed, in any way?
3. What problems, if any, were experienced during procurement of DFS? How were these addressed?
4. Could the procurement process be further improved in any way?

DFS disbursal (program/retail)

1. What is the process of disbursal of DFS to Fair Price Shops/Anganwadi centres? (requirement, allocation, delivery, receipt, stocks)
2. How is this process monitored to ensure regularity of supply? What is done with unused/expired stock?
3. What problems, if any, were experienced during disbursal of DFS to Fair Price Shops/Anganwadi centres? (use of hooks for unloading sacks of DFS)
4. How were these addressed?
5. Could the DFS disbursal process be further improved in any way?

DFS distribution (household)

1. What is the eligibility criteria for households and what is the entitlement? Is it adequate? An average household of 5 members is estimated to require at least 1.5 kg salt per month, if the allocation is 1 kg DFS per household per month, is there any plan to raise the entitlement?
2. How is uptake of DFS salt by households monitored? Did the uptake change over the course of the project? If yes, how was it ensured/addressed?
3. How is quality of DFS ensured upto the household level?
4. Is there a grievance redressal mechanism? What are the main grievances that have been reported? How have these been addressed?

Behavior change/DFS use (households)

1. What were the challenges in getting households to switch from their usual salt to DFS? How have these changed over time? ( for ex: with increasing awareness)
2. What was done to address these challenges?
3. How well did these efforts address household concerns?

Monitoring/evaluation

1. How is DFS implementation monitored? (Govt system and NI system- differences, similarities, co-ordination, collaboration)
2. How effective is the monitoring system for DFS implementation? How can it be improved?
3. What other studies have been conducted to understand DFS implementation? How were their findings incorporated?
4. What plans does the government have to evaluate this project?

NI’s Role

1. How has NI supported you in DFS introduction and implementation in the state? (NI value add)
2. What further support would you like NI to provide for DFS implementation?
3. Do you have any feedback for NI staff on how they could improve their work?
4. **Wrap-up**
5. Overall, what was your impression of the introduction of DFS to ICDS/PDS?
6. What do you consider the top 3 challenges?
7. What do you consider the top 3 successes?
8. If another state were to introduce DFS to a social welfare scheme, what would your recommendations be?
9. Is there anything else you’d like to mention?

***

**Process evaluation of the introduction of double fortified salt in pilot districts in Madhya Pradesh and Gujarat, India**

**In-depth Interview (IDI) with Regulators**

**Date:** [date]

**Prompts**

- At start:
  - Read consent
  - Ask respondent if the interview can be recorded
  - Ask if there is an obligation at the top of the hour
- At end:
  - Is there anyone else that they think I should speak with?
  - Thank them for their time.

**Section – I: Background Information**

1. Government Official name ______________________________________________

Designation _______________________________________________

Department _______________________________________________

1. Role within project ______________________________________________
2. Time frame involved ______________________________________________

**Section – II: Topic Guide for IDI**

1. **Background**
2. Can you please tell me about your role in DFS regulation in India?

(did this role stay consistent/change over the lifetime of your interaction?)

1. Who else did you work closely with on this project?

(what were their responsibilities?)

1. Can you please describe a timeline of how the project in [state] rolled out?
2. **Activities/Tasks (Note – questions for specific sections only asked of individuals who had roles/activities related to those sections. E.g. a staff member who did not interface or work on advocacy with policymakers would not be asked questions under that section)**

Development of DFS standards and guidelines

1. What was the national context in which the regulation on fortified foods, especially double fortified salt was notified in 2018? (scientific breakthrough, policy push?)
2. For how long had such regulation been under consideration of FSSAI and the MOHFW?
3. What were the processes through which the ‘Fortification of Foods’ Regulation was drafted? (consultations, committee etc?)
4. What were the processes through which DFS standards were developed? What were the criteria considered in accepting both DFS formulations?
5. What were the processes through which the ‘Fortification of Foods’ Regulation was approved?
6. What kind of feedback/response has the regulation received?
7. Are there amendments planned and/or needed?

Scale up of DFS through ICDS/PDS in Gujarat/Madhya Pradesh

1. What is FSSAI’s role in state implementation of DFS through social safety net programmes such as in Madhya Pradesh and Gujarat?
2. Which other states are engaged in similar interventions?
3. What has worked well in these interventions?
4. How could these efforts be further improved?

Behavior change/DFS use

1. How is FSSAI promoting the shift to DFS by households?
2. What have been the challenges in facilitating this shift? How have these challenges been addressed?
3. How could these efforts be further improved?

Monitoring/evaluation

1. How is FSSAI monitoring the quality of DFS manufactured by salt producers?
2. What kind of challenges have emerged in such monitoring?
3. How have these been addressed?
4. What has worked well in ensuring quality of DFS?

NI’s Role

1. How has NI supported FSSAI in DFS regulation?
2. What further support would you like NI to provide for DFS implementation?
3. Do you have any feedback for NI staff on how they could improve their work?
4. **Wrap-up**
5. Overall, what was your impression of the introduction of DFS to ICDS/PDS?
6. What do you consider the top 3 challenges?
7. What do you consider the top 3 successes?
8. If another state were to introduce DFS to a social welfare scheme, what would your recommendations be?
9. Is there anything else you’d like to mention?

**For State level**

**Food and Drug Control Administration (FDCA) Officials**

1. What is the FDCA’s role in regulating the introduction of DFS in social safety net programmes at the state level?
2. What is the FDCA’s role in regulating the quality of food products supplied as a part of social safety net programmes such as the ICDS and the PDS?
3. Is the state level FDCA currently performing the role of monitoring DFS quality standards?
4. If yes, what are the processes through which DFS quality is monitored from the factory to the household level?
5. If no, why is FDCA not performing its role? What are the challenges experienced?
6. Who is monitoring the quality of DFS supplied through social safety net programmes?
7. How does FDCA collaborate/coordinate with them?
8. How does FDCA collaborate/co-ordinate with FSSAI?
9. Was FDCA involved in developing DFS quality standards?
10. How has NI supported the FDCA at the state level?

**‘Process evaluation of the introduction of double fortified salt in pilot districts in Madhya Pradesh and Gujarat, India**

**In-depth Interview (IDI) with Salt Manufacturers**

**Date:** [date]

**Prompts**

- At start:
  - Read consent
  - Ask respondent if the interview can be recorded
  - Ask if there is an obligation at the top of the hour
- At end:
  - Is there anyone else that they think I should speak with?
  - Thank them for their time.

**Section – I: Background Information**

1. Respondent name ______________________________________________

Designation _______________________________________________

Department _______________________________________________

1. Role within project ______________________________________________
2. Time frame involved ______________________________________________

**Section – II: Topic Guide for IDI**

1. **Background**
2. Can you please tell me about your role within the DFS implementation project?

(did this role stay consistent/change over the lifetime of your interaction?)

1. Who else did you/ your company work closely with on this project?

(what were their responsibilities?)

1. Can you please describe a timeline of when your company got involved in this project?
2. **Activities/Tasks (Note – questions for specific sections only asked of individuals who had roles/activities related to those sections. E.g. a staff member who did not interface or work on advocacy with policymakers would not be asked questions under that section)**

Bidding/applying for DFS supply

1. How did you come to know about the plan to introduce DFS in ICDS/PDS?
2. Were you already engaged in supplying/producing iodised salt to ICDS/PDS? If yes, what was the difference in processes you were required to do/follow to produce and distribute DFS?
3. Is any specific technical capacity required to bid/apply for DFS manufacture? Did you already have that capacity of did you have to build/acquire it?
4. What steps did the tendering process involve?
5. How long did the tendering process take?
6. What proportion, does this contract constitute of your total manufacture and sale of salt?
7. Is this a profitable contract for you? Will you apply for future contracts as well?

Iron/DFS manufacture

1. What kind of iron formulation do you use for DFS manufacture? Was the formulation specified in the contract? Is this the most suitable formulation for fortification?
2. Do you manufacture the iron formulation or purchase it? If purchase, where do you purchase it from? How was the supply source selected?
3. Can you describe the DFS manufacturing process?
4. Is there any way in which the iron formulation manufacture/ procurement process can be improved?
5. Is there any way in which the DFS manufacture process can be improved?

DFS packaging

1. What are the packaging specifications for DFS? Are they the same or different from iodised salt?
2. How are the specifications monitored?
3. What are the labelling specifications for the packaging? In which languages?
4. Have any concerns ever been raised about the packaging and labelling?
5. How were these addressed?
6. Is there any way in which the packaging and labelling of DFS can be improved?

DFS quality

1. What kind of salt was used to create DFS? (list all)
2. What are the appropriate iron and iodine levels that should be in salt when sold to households? What is the shelf life of DFS? How is quality monitored throughout the DFS manufacturing process?
3. Does the government check each batch supplied for quality independently? If yes, how?
4. Have any concerns been ever raised about the quality of DFS? (what kind of concerns? By whom?)
5. How were these concerns addressed?

DFS supply & delivery

1. How is DFS supply organized? Is it to a centralized point or directly to Fair Price Shops/Anganwadi Centres?
2. What is the frequency and quantity of supply? How is this determined?
3. How is supply and delivery monitored?
4. Has supply ever been delayed? If yes, why? How was the problem resolved?
5. What are the challenges in ensuring regular supply and timely delivery?
6. What happens if a consignment is damaged or delivery refused? Has this ever happened? If yes, what happened and how was it adjusted/resolved?
7. How can DFS supply and delivery be further streamlined?

DFS supply finances

1. How is payment processed? What is the frequency of raising invoices?
2. How are invoices checked? What questions have been raised on invoices?
3. What is the average time taken to receive a payment?
4. Is the payment received the same as the invoice raised ? If there are differences, why do these come up?
5. What is the maximum delay experienced in receiving payments?
6. How can payment processing be made more efficient?

Monitoring/evaluation

1. Are govt officials expected to conduct monitoring visits to the manufacturing facility as per the contract?
2. If, yes how many visits have been conducted till date and with what frequency?
3. What generally happens on a visit?
4. What kind of suggestions have been made by officials? How have these been incorporated?

NI’s role

1. Has NI supported you in any way in DFS manufacture and supply to ICDS/PDS? What role has it played?
2. Has NI’s role been helpful?
3. In what way can NI’s support be improved?
4. **Wrap-up**
5. Overall, what was your impression of the introduction of DFS to ICDS/PDS?
6. What do you consider the top 3 challenges?
7. What do you consider the top 3 successes?
8. If another state were to introduce DFS to a social welfare scheme, what would your recommendations be?
9. Is there anything else you’d like to mention?

***

**Process evaluation of the introduction of double fortified salt in pilot districts in Madhya Pradesh and Gujarat, India**

**In-depth Interview (IDI) with Nutrition International technical support staff**

**Date:** [date]

**Prompts**

- At start:
  - Read consent
  - Ask respondent if the interview can be recorded
  - Ask if there is an obligation at the top of the hour
- At end:
  - Is there anyone else that they think I should speak with?
  - Thank them for their time.

**Section – I: Background Information**

1. Staff name ______________________________________________
2. Role within project ______________________________________________

(and current position, state)

1. Time frame involved ______________________________________________

**Section – II: Topic Guide for IDI**

1. **Background**
2. Can you please tell me about your role within the project?

(did this role stay consistent/change over the lifetime of your interaction?)

1. Who else did you work closely with at NI on this project?

(what were their responsibilities?)

| **Category** | **Involvement?** |
| --- | --- |
| **Iron/DFS procurement** |  |
| **DFS quality** |  |
| **DFS procurement** |  |
| **DFS distribution (retail)** |  |
| **DFS distribution (household)** |  |
| **Advocacy** |  |
| **Behavior change** |  |
| **Monitoring/evaluation** |  |
| **NI strategy** |  |

1. In what specific ways did NI provide support to both the state/national governments? (what global support was necessary/relevant to apply to India?)
2. Can you please describe a timeline of the project? (eg State-level activities, or national-level activities involving regulatory framework)
3. **Activities/Tasks (Note – questions for specific sections only asked of individuals who had roles/activities related to those sections. E.g. a staff member who did not interface or work on advocacy with policymakers would not be asked questions under that section)**

Iron formulation/DFS procurement

1. What kind of iron formulation was used in the DFS in [state, program]?
2. Can you describe the process to create DFS? (e.g., did the salt producer produce the iron formulation internally or did they purchase the iron formulation and then blend into their salt?)
3. Where was the DFS used in [state, program] purchased from? (How did procurement shift from iodized salt to DFS? Did the DFS source change over the project’s timeline?)
4. Who made the decisions regarding where to procure DFS?
5. How was a DFS manufacturer selected (e.g. based on what parameters? Price?)
6. In hindsight, is there anything you would have changed about the iron formulation/DFS procurement process?
7. What do you think worked well in the iron formulation/DFS procurement process?
8. What did not work well in the iron formulation/DFS procurement process?
9. What kind of support did salt industry need/request from NI to procure the iron formulation?
10. What kind of support did the government need to procure DFS?

DFS quality

1. What kind of salt was used to create DFS? (list all)
2. Were there any concerns about the quality of DFS? (what kind of concerns? By who?)
3. How did [NI, DFS producer, state government] try to address those concerns?
4. Do you feel that those concerns were addressed adequately?
5. What processes were in place to ensure that the appropriate iron and iodine levels were in DFS by the time that households purchased the salt?

DFS procurement

1. Can you describe the process that the government used to procure DFS for [ICDS/PDS]?

(responsible entities? Storage condition, length? Was adequate lead time allowed to ensure consistent stock within ICDS/PDS? What was NI’s involvement?)

1. What do you think worked well in the procurement process?
2. What do you think did not work well in the procurement process?

DFS distribution (program/retail)

1. After the government procured DFS from manufacturers, how was it then distributed to fair price shops/Angawadi centres? (timeline, ordering mechanism, how were volumes decided?, NI’s role)
2. What do you think worked well in the distribution process?
3. What do you think did not work well in the distribution process? (diversion?)

DFS distribution (household)

1. How was DFS sold/provided to households? (did this differ compared to iodized salt, in terms of packaging, appearance, price?)
2. How successful do you think the project was in getting households to purchase and also consume DFS? (of the households that did purchase, why did they purchase?)
3. In hindsight, would you change anything about how DFS was sold/provided to households?

Advocacy (policymakers)

1. In what ways did NI provide advocacy support to the government? (what was NI’s role in developing the DFS standard?)
2. Can you describe how [state] decided to add DFS to ICDS/PDS? (what was NI’s role? Who/what were other persuasive entities? What were key activities/arguments/considerations? Who was the key decision-maker?)
3. How did [state] make decisions around budget allocation to implement DFS? (will these be sustained?)
4. What do you think worked well in advocating to the government to add DFS?
5. What do you think did not work well in advocating to the government to add DFS?

Behavior change/DFS use (households)

1. What were challenges in getting households to switch from their usual salt to DFS? (e.g., cost differences? Color? Taste?)
2. What was done [by NI, manufacturers, state government] to address these challenges?
3. How well did these efforts address household concerns?
4. In hindsight, is there anything that you would have done differently to try and change household behavior or use of DFS?

Monitoring/evaluation

1. Can you describe the processes NI put in place to monitor and evaluate the implementation of DFS? (is there anything that you think should have been done differently? What worked well?)
2. Can you describe the processes the government put in place to monitor and evaluate the implementation of DFS? (is there anything that you think should have been done differently? What worked well?)

NI internal strategy

1. Can you describe NI’s strategy towards DFS in India? (how were these states and programs identified/selected? Is there a national strategy?)
2. How does NI develop or encourage support for fortification within the government of India (both national and state)?
3. How do you think NI’s strategy is similar to other partners who advocate for food fortification in India?
4. How do you think NI’s strategy is different to other partners who advocate for food fortification in India?
5. In what ways do you think NI is successful in its food fortification efforts in India?
6. In what ways do you think NI has not been as successful in its food fortification efforts in India?
7. **Wrap-up**
8. Overall, what was your impression of the introduction of DFS to ICDS/PDS?
9. What do you consider the top 3 challenges? (prompt for mention of titanium dioxide if not mentioned earlier)
10. What do you consider the top 3 successes?
11. If another state were to introduce DFS to a social welfare scheme, what would your recommendations be?
12. Is there anything else you’d like to mention?

***
